# Methyl 4-[(5-chloro­pyrimidin-2-yl)carbamo­yl]benzoate

**DOI:** 10.1107/S1600536811025268

**Published:** 2011-07-02

**Authors:** Chun-Hsiang Lu, Chia-Jun Wu, Chun-Wei Yeh, Jhy-Der Chen

**Affiliations:** a Department of Chemistry, Chung-Yuan Christian University, Chung-Li, Taiwan

## Abstract

Mol­ecules of the title compound, C_13_H_10_ClN_3_O_3_, form centrosymmetric dimers *via* inter­molecular N—H⋯N hydrogen bonds generating an *R*_2_^2^(8) motif. The dimers are further connected through an O⋯Cl—C halogen bond [O⋯Cl = 3.233 (1) Å and O⋯Cl—C = 167.33 (1)°] into a chain along [110]. The secondary amide group adopts a *cis* conformation. Weak C—H⋯N hydrogen bonds among the methyl benzoate and pyrimidyl rings are also observed in the crystal structure.

## Related literature

For silver complexes of the title compound, see: Wu *et al.* (2011 ▶). For the conformation of the amide group in similar compounds, see: Forbes *et al.* (2001 ▶); Oertli *et al.* (1992 ▶).
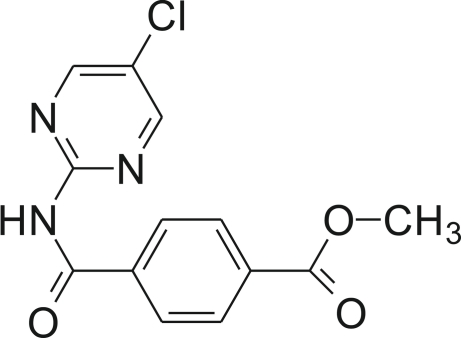


## Experimental

### 

#### Crystal data

C_13_H_10_ClN_3_O_3_
*M* *_r_* = 291.69
Triclinic, 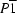

*a* = 5.9068 (8) Å
*b* = 7.3378 (9) Å
*c* = 15.816 (4) Å
α = 78.259 (14)°
β = 82.030 (13)°
γ = 67.986 (10)°
*V* = 620.78 (19) Å^3^
*Z* = 2
Mo *K*α radiation
μ = 0.32 mm^−1^
*T* = 295 K
0.5 × 0.4 × 0.3 mm

#### Data collection

Siemens P4 diffractometer
Absorption correction: ψ scan (*XSCANS*; Siemens, 1995 ▶) *T*_min_ = 0.888, *T*_max_ = 0.918
2811 measured reflections
2140 independent reflections
1715 reflections with *I* > 2σ(*I*)
*R*_int_ = 0.026
3 standard reflections every 97 reflections  intensity decay: none

#### Refinement

*R*[*F* ^2^ > 2σ(*F* ^2^)] = 0.036
*wR*(*F* ^2^) = 0.098
*S* = 1.02
2140 reflections
182 parameters
H-atom parameters constrained
Δρ_max_ = 0.17 e Å^−3^
Δρ_min_ = −0.21 e Å^−3^

### 

Data collection: *XSCANS* (Siemens, 1995 ▶); cell refinement: *XSCANS*; data reduction: *XSCANS*; program(s) used to solve structure: *SHELXS97* (Sheldrick, 2008 ▶); program(s) used to refine structure: *SHELXL97* (Sheldrick, 2008 ▶); molecular graphics: *SHELXTL* (Sheldrick, 2008 ▶); software used to prepare material for publication: *SHELXTL*.

## Supplementary Material

Crystal structure: contains datablock(s) I, global. DOI: 10.1107/S1600536811025268/gk2391sup1.cif

Structure factors: contains datablock(s) I. DOI: 10.1107/S1600536811025268/gk2391Isup2.hkl

Supplementary material file. DOI: 10.1107/S1600536811025268/gk2391Isup3.cml

Additional supplementary materials:  crystallographic information; 3D view; checkCIF report

## Figures and Tables

**Table 1:** Hydrogen-bond geometry (Å, °)

*D*—H⋯*A*	*D*—H	H⋯*A*	*D*⋯*A*	*D*—H⋯*A*
N3—H3*B*⋯N2^i^	0.86	2.10	2.959 (2)	176
C13—H13*C*⋯N1^ii^	0.96	2.57	3.339 (2)	138

Symmetry codes: (i) 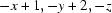; (ii) 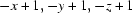.

**Fig. 1.**
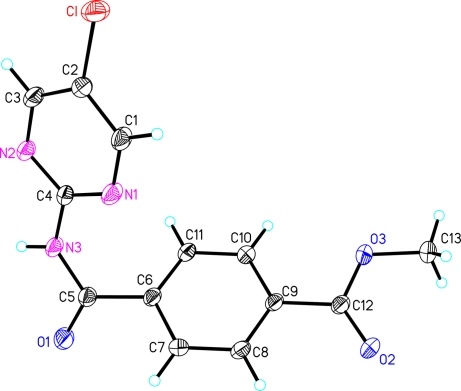
Crystal structure of the title compound wiFig. 1. Crystal structure of the title compound with atom labeling and displacement ellipsoids drawn at the 30% probability level.

**Fig. 2.**
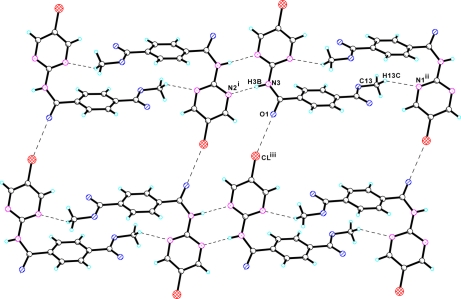
Partial packing diagram showing C—H···N and N—H···N hydrogen bonds and C—Cl···O interactions among the molecules, with atom labeling and displacement ellipsoids drawn at the 30% probability level. Symmetric code: (i) 1 - x, 2 - y, -z; (ii) 1 - x, 1 - y, 1 - z; (iii) 1 + x, 1 + y, z.

## supplementary crystallographic information

### Comment

The silver(I) complex containg methyl 4-(5-chloropyrimidin-2-ylcarbamoyl)benzoate ligand has been reported, which shows a two-dimensional network (Wu *et al.*, 2011). Within this project the crystal structure of the title compound was determined (Fig. 1). In its crystal structure intermolecular N—H···N hydrogen bonds are found (Table 1) and the molecules are also interlinked through C—Cl···O interactions [3.233 (1) Å and 167.33 (1)°]. Weak C—H···N hydrogen bonds among the methyl benzoate and pyrimidyl rings are also observed in the solid state (Fig. 2). In the crystal structure the amide group of the title compound adopts the *cis* conformation, which is in marked contrast to the *trans* conformation found in the Ag complex (Wu *et al.*, 2011).

### Experimental

The title compound was prepared according to a published procedure (Wu *et al.*, 2011). Block crystals suitable for X-ray crystallography were obtained by slow evaporation of the solvent from a solution of the title compound in methanol.

### Refinement

H atoms bound to C and N atoms were placed in idealized positions and constrained to ride on their parent atoms, with C—H = 0.93 - 0.96Å and N—H = 0.86 Å, and with *U*_iso_(H) = 1.2 or 1.5 *U*_eq_(C/N).

### Crystal data

**Table:**

C_13_H_10_ClN_3_O_3_	*Z* = 2
*M**_r_* = 291.69	*F*(000) = 300
Triclinic, *P*1	*D*_x_ = 1.560 Mg m^−^^3^
Hall symbol: -P 1	Mo *K*α radiation, λ = 0.71073 Å
*a* = 5.9068 (8) Å	Cell parameters from 28 reflections
*b* = 7.3378 (9) Å	θ = 4.7–13.3°
*c* = 15.816 (4) Å	µ = 0.32 mm^−^^1^
α = 78.259 (14)°	*T* = 295 K
β = 82.030 (13)°	Block, colourless
γ = 67.986 (10)°	0.5 × 0.4 × 0.3 mm
*V* = 620.78 (19) Å^3^	

### Data collection

**Table:**

Siemens P4 diffractometer	1715 reflections with *I* > 2σ(*I*)
Radiation source: fine-focus sealed tube	*R*_int_ = 0.026
graphite	θ_max_ = 25.0°, θ_min_ = 2.6°
ω scans	*h* = −1→7
Absorption correction: ψ scan (*XSCANS*; Siemens, 1995)	*k* = −8→8
*T*_min_ = 0.888, *T*_max_ = 0.918	*l* = −18→18
2811 measured reflections	3 standard reflections every 97 reflections
2140 independent reflections	intensity decay: none

### Refinement

**Table:**

Refinement on *F*^2^	Primary atom site location: structure-invariant direct methods
Least-squares matrix: full	Secondary atom site location: difference Fourier map
*R*[*F*^2^ > 2σ(*F*^2^)] = 0.036	Hydrogen site location: inferred from neighbouring sites
*wR*(*F*^2^) = 0.098	H-atom parameters constrained
*S* = 1.02	*w* = 1/[σ^2^(*F*_o_^2^) + (0.0507*P*)^2^ + 0.1938*P*] where *P* = (*F*_o_^2^ + 2*F*_c_^2^)/3
2140 reflections	(Δ/σ)_max_ < 0.001
182 parameters	Δρ_max_ = 0.17 e Å^−^^3^
0 restraints	Δρ_min_ = −0.21 e Å^−^^3^

### Special details

**Table:**

Experimental. Refinement of *F*^2^ against ALL reflections. The weighted *R*-factor *wR* and goodness of fit *S* are based on *F*^2^, conventional *R*-factors *R* are based on *F*, with *F* set to zero for negative *F*^2^. The threshold expression of *F*^2^ > σ(*F*^2^) is used only for calculating *R*-factors(gt) *etc*. and is not relevant to the choice of reflections for refinement. *R*-factors based on *F*^2^ are statistically about twice as large as those based on *F*, and *R*- factors based on ALL data will be even larger.
Geometry. All e.s.d.'s (except the e.s.d. in the dihedral angle between two l.s. planes) are estimated using the full covariance matrix. The cell e.s.d.'s are taken into account individually in the estimation of e.s.d.'s in distances, angles and torsion angles; correlations between e.s.d.'s in cell parameters are only used when they are defined by crystal symmetry. An approximate (isotropic) treatment of cell e.s.d.'s is used for estimating e.s.d.'s involving l.s. planes.
Refinement. Refinement of *F*^2^ against ALL reflections. The weighted *R*-factor *wR* and goodness of fit *S* are based on *F*^2^, conventional *R*-factors *R* are based on *F*, with *F* set to zero for negative *F*^2^. The threshold expression of *F*^2^ > σ(*F*^2^) is used only for calculating *R*-factors(gt) *etc*. and is not relevant to the choice of reflections for refinement. *R*-factors based on *F*^2^ are statistically about twice as large as those based on *F*, and *R*- factors based on ALL data will be even larger.

### Fractional atomic coordinates and isotropic or equivalent isotropic displacement parameters (Å^2^)

**Table:**

	*x*	*y*	*z*	*U*_iso_*/*U*_eq_	
Cl	0.10131 (12)	0.38338 (9)	0.11742 (4)	0.0550 (2)	
C1	0.3775 (4)	0.5274 (3)	0.18741 (12)	0.0397 (5)	
H1A	0.3782	0.4287	0.2349	0.048*	
C2	0.2528 (4)	0.5446 (3)	0.11729 (12)	0.0367 (5)	
C3	0.2556 (4)	0.6923 (3)	0.04841 (13)	0.0431 (5)	
H3A	0.1746	0.7055	−0.0001	0.052*	
C4	0.4859 (4)	0.7890 (3)	0.12062 (11)	0.0325 (4)	
C5	0.7163 (4)	0.9541 (3)	0.18183 (12)	0.0347 (4)	
C6	0.6516 (4)	0.8916 (3)	0.27530 (11)	0.0318 (4)	
C7	0.8388 (4)	0.8014 (3)	0.33151 (12)	0.0359 (5)	
H7A	1.0007	0.7763	0.3104	0.043*	
C8	0.7837 (4)	0.7492 (3)	0.41870 (12)	0.0363 (5)	
H8A	0.9091	0.6881	0.4562	0.044*	
C9	0.5424 (4)	0.7875 (3)	0.45062 (11)	0.0311 (4)	
C10	0.3554 (4)	0.8838 (3)	0.39472 (12)	0.0347 (4)	
H10A	0.1930	0.9141	0.4162	0.042*	
C11	0.4102 (4)	0.9345 (3)	0.30739 (12)	0.0354 (5)	
H11A	0.2848	0.9976	0.2701	0.043*	
C12	0.4893 (4)	0.7203 (3)	0.54431 (12)	0.0331 (4)	
C13	0.1836 (4)	0.6842 (3)	0.65241 (12)	0.0411 (5)	
H13A	0.0194	0.6854	0.6568	0.062*	
H13B	0.1925	0.7730	0.6878	0.062*	
H13C	0.2932	0.5516	0.6718	0.062*	
N1	0.4973 (3)	0.6482 (3)	0.18929 (10)	0.0408 (4)	
N2	0.3709 (3)	0.8174 (3)	0.04906 (10)	0.0393 (4)	
N3	0.6073 (3)	0.9194 (2)	0.11818 (10)	0.0378 (4)	
H3B	0.6161	0.9910	0.0684	0.045*	
O1	0.8550 (3)	1.0460 (3)	0.16089 (9)	0.0527 (4)	
O2	0.6419 (3)	0.6464 (3)	0.59689 (9)	0.0512 (4)	
O3	0.2527 (3)	0.7486 (2)	0.56330 (8)	0.0406 (4)	

### Atomic displacement parameters (Å^2^)

**Table:**

	*U*^11^	*U*^22^	*U*^33^	*U*^12^	*U*^13^	*U*^23^
Cl	0.0698 (4)	0.0616 (4)	0.0504 (3)	−0.0436 (3)	−0.0186 (3)	0.0026 (3)
C1	0.0561 (14)	0.0391 (11)	0.0274 (10)	−0.0232 (10)	−0.0099 (9)	0.0038 (8)
C2	0.0417 (12)	0.0423 (11)	0.0315 (10)	−0.0212 (10)	−0.0039 (9)	−0.0052 (8)
C3	0.0539 (13)	0.0585 (13)	0.0267 (10)	−0.0312 (11)	−0.0121 (9)	−0.0006 (9)
C4	0.0379 (11)	0.0393 (10)	0.0220 (9)	−0.0168 (9)	−0.0034 (8)	−0.0017 (7)
C5	0.0418 (11)	0.0381 (11)	0.0285 (10)	−0.0203 (9)	−0.0041 (8)	−0.0023 (8)
C6	0.0418 (11)	0.0350 (10)	0.0255 (9)	−0.0210 (9)	−0.0059 (8)	−0.0036 (8)
C7	0.0319 (11)	0.0478 (12)	0.0332 (10)	−0.0200 (9)	−0.0044 (8)	−0.0057 (8)
C8	0.0382 (11)	0.0443 (12)	0.0299 (10)	−0.0183 (9)	−0.0122 (8)	−0.0003 (8)
C9	0.0369 (11)	0.0347 (10)	0.0264 (9)	−0.0172 (9)	−0.0080 (8)	−0.0031 (7)
C10	0.0333 (11)	0.0424 (11)	0.0292 (10)	−0.0150 (9)	−0.0048 (8)	−0.0028 (8)
C11	0.0375 (12)	0.0425 (11)	0.0265 (10)	−0.0149 (9)	−0.0104 (8)	0.0007 (8)
C12	0.0392 (12)	0.0347 (10)	0.0287 (10)	−0.0165 (9)	−0.0085 (9)	−0.0024 (8)
C13	0.0477 (13)	0.0498 (12)	0.0272 (10)	−0.0225 (10)	−0.0024 (9)	0.0007 (9)
N1	0.0575 (11)	0.0432 (10)	0.0283 (9)	−0.0268 (9)	−0.0143 (8)	0.0047 (7)
N2	0.0504 (11)	0.0522 (11)	0.0224 (8)	−0.0285 (9)	−0.0084 (7)	0.0016 (7)
N3	0.0519 (11)	0.0469 (10)	0.0217 (8)	−0.0287 (9)	−0.0091 (7)	0.0045 (7)
O1	0.0702 (11)	0.0736 (11)	0.0343 (8)	−0.0522 (10)	−0.0029 (7)	−0.0013 (7)
O2	0.0452 (9)	0.0793 (11)	0.0298 (8)	−0.0269 (8)	−0.0155 (7)	0.0074 (7)
O3	0.0394 (8)	0.0553 (9)	0.0254 (7)	−0.0196 (7)	−0.0060 (6)	0.0043 (6)

### Geometric parameters (Å, °)

**Table:**

Cl—C2	1.730 (2)	C7—H7A	0.9300
C1—N1	1.332 (3)	C8—C9	1.387 (3)
C1—C2	1.374 (3)	C8—H8A	0.9300
C1—H1A	0.9300	C9—C10	1.390 (3)
C2—C3	1.375 (3)	C9—C12	1.492 (3)
C3—N2	1.334 (3)	C10—C11	1.382 (3)
C3—H3A	0.9300	C10—H10A	0.9300
C4—N1	1.328 (2)	C11—H11A	0.9300
C4—N2	1.341 (2)	C12—O2	1.206 (2)
C4—N3	1.387 (2)	C12—O3	1.334 (2)
C5—O1	1.217 (2)	C13—O3	1.446 (2)
C5—N3	1.378 (3)	C13—H13A	0.9600
C5—C6	1.497 (3)	C13—H13B	0.9600
C6—C11	1.385 (3)	C13—H13C	0.9600
C6—C7	1.391 (3)	N3—H3B	0.8600
C7—C8	1.381 (3)		
			
N1—C1—C2	121.82 (17)	C8—C9—C10	119.59 (17)
N1—C1—H1A	119.1	C8—C9—C12	119.08 (17)
C2—C1—H1A	119.1	C10—C9—C12	121.31 (17)
C1—C2—C3	117.43 (19)	C11—C10—C9	120.16 (18)
C1—C2—Cl	120.22 (15)	C11—C10—H10A	119.9
C3—C2—Cl	122.34 (16)	C9—C10—H10A	119.9
N2—C3—C2	122.04 (18)	C10—C11—C6	120.13 (18)
N2—C3—H3A	119.0	C10—C11—H11A	119.9
C2—C3—H3A	119.0	C6—C11—H11A	119.9
N1—C4—N2	126.16 (18)	O2—C12—O3	123.57 (18)
N1—C4—N3	119.57 (16)	O2—C12—C9	124.38 (19)
N2—C4—N3	114.23 (16)	O3—C12—C9	112.05 (16)
O1—C5—N3	118.93 (17)	O3—C13—H13A	109.5
O1—C5—C6	120.70 (17)	O3—C13—H13B	109.5
N3—C5—C6	120.25 (17)	H13A—C13—H13B	109.5
C11—C6—C7	119.85 (17)	O3—C13—H13C	109.5
C11—C6—C5	121.36 (17)	H13A—C13—H13C	109.5
C7—C6—C5	118.62 (18)	H13B—C13—H13C	109.5
C8—C7—C6	119.92 (19)	C4—N1—C1	116.57 (16)
C8—C7—H7A	120.0	C3—N2—C4	115.95 (16)
C6—C7—H7A	120.0	C5—N3—C4	131.10 (16)
C7—C8—C9	120.30 (18)	C5—N3—H3B	114.4
C7—C8—H8A	119.9	C4—N3—H3B	114.4
C9—C8—H8A	119.9	C12—O3—C13	116.21 (15)

### Hydrogen-bond geometry (Å, °)

**Table:**

*D*—H···*A*	*D*—H	H···*A*	*D*···*A*	*D*—H···*A*
N3—H3B···N2^i^	0.86	2.10	2.959 (2)	176
C13—H13C···N1^ii^	0.96	2.57	3.339 (2)	138

Symmetry codes: (i) −*x*+1, −*y*+2, −*z*; (ii) −*x*+1, −*y*+1, −*z*+1.
